# Structure and NMR properties of the dinuclear complex di-μ-azido-κ^4^ *N* ^1^:*N* ^1^-bis­[(azido-κ*N*)(pyridine-2-carboxamide-κ^2^ *N* ^1^,*O*)zinc(II)]

**DOI:** 10.1107/S2056989020016680

**Published:** 2021-01-08

**Authors:** Cándida Pastor Ramírez, Sylvain Bernès, Samuel Hernández Anzaldo, Yasmi Reyes Ortega

**Affiliations:** a Instituto de Ciencias, Benemérita Universidad Autónoma de Puebla, Av. San Claudio y 18 Sur, 72570 Puebla, Pue., Mexico; b Instituto de Física, Benemérita Universidad Autónoma de Puebla, 72570 Puebla, Pue., Mexico

**Keywords:** crystal structure, azido bridge, coordination compound, zinc, picolinamide

## Abstract

The new diamagnetic complex, [Zn_2_(N_3_)_4_(C_6_H_6_N_2_O)_2_] or [Zn_2_(pca)_2_(μ_1,1_-N_3_)_2_(N_3_)_2_] was synthesized using pyridine-2-carboxamide (pca) and azido ligands, and characterized using various techniques: IR spectroscopy and single-crystal X-ray diffraction in the solid state, and nuclear magnetic resonance (NMR) in solution. The mol­ecule is placed on an inversion centre in space group *P* . The pca ligand chelates the metal centre *via* the pyridine N atom and the carbonyl O atom. One azido ligand bridges the two symmetry-related Zn^2+^ cations in the *end-on* coordination mode, while the other independent azido anion occupies the fifth coordination site, as a terminal ligand. The resulting five-coordinate Zn centres have a coordination geometry inter­mediate between trigonal bipyramidal and square pyramidal. The behaviour of the title complex in DMSO solution suggests that it is a suitable NMR probe for similar or isostructural complexes including other transition-metal ions. The diamagnetic nature of the complex is reflected in similar ^1^H and ^13^C NMR chemical shifts for the free ligand pca as for the Zn complex.

## Abstract 2

The crystal structure of a dimeric Zn^II^ complex with picolinamide and azido ligands is characterized in the solid state and in solution.

## Chemical context   

Polynuclear complexes have received the attention of coordination chemists as they are ideal candidates for developing new functional mol­ecular materials. In the design and preparation of such systems, a number of synthetic strategies have been used for propagating new motifs, affording a large number of polynuclear complexes with potential applications (Miller & Drillon, 2002 ▸). Complexes based on Zn^2+^ ions are of inter­est because of the versatility of this transition metal towards different kinds of chelating ligands, and its ability to bind ligands with different coordination numbers, ranging from two to six (Sakai *et al.*, 2006 ▸). Some complexes have been proposed as models for the active sites of zinc-containing enzymes (Parkin, 2000 ▸; Döring *et al.*, 2002 ▸), while others have been studied for their catalytic properties (Dey *et al.*, 2002 ▸) or for the purpose of producing OLED devices (Sano *et al.*, 2000 ▸; Tokito *et al.*, 2000 ▸; Ray *et al.*, 2012 ▸).

Upon coordination of a ligand to a metal centre, the ligand properties, such as electrophilic or nucleophilic character, acidity, susceptibility to oxidation or reduction, can be significantly altered, thereby enhancing or inhibiting its reactivity (Konidaris *et al.*, 2012 ▸). Co-ligands are also important for the structure and properties of the complex, especially if they can bridge metal centres. Among them, the azido ligand, N_3_^−^, has been widely used in the building of mol­ecular magnetic materials with a rich diversity of topologies (Ribas *et al.*, 1999 ▸; Hong & Chen, 2009 ▸). The challenging aspect of N_3_^−^ is its great coordination flexibility, which turns out to be rather a drawback since structures are poorly predictable. However, the correlation between the structures of polynuclear complexes including azido bridges and their magnetic properties is now well understood (Husain *et al.*, 2012 ▸; Yu *et al.*, 2007 ▸).

The azido ion can link two or more metal ions in different configurations. The most representative are the *end-to-end* (EE) mode, in which two terminal N atoms bridge the metals, and the *end-on* (EO) mode, in which only one terminal N atom is used (Dori & Ziolo, 1973 ▸; Mautner *et al.*, 2013 ▸). Based on a survey of the CSD (Groom *et al.*, 2016 ▸), the prevalence of the EO mode is much higher than the EE mode, by a factor of about ten. Mixed species having both terminal (*i.e*. non-bridg­ing) and EE/EO bridging azides are known, but are not so common. Several architectures occur depending on whether EE or EO bridges are present, which can be symmetric or asymmetric, single or multiple, and associated or not with other bridges (Goher *et al.*, 2000 ▸; Maji *et al.*, 2001 ▸).

In this context, our group has paid attention to the synthesis of Zn^2+^ complexes including azido ligands, with the aim of using these diamagnetic compounds as NMR probes for other structurally related or analogous complexes. Herein, we report the mol­ecular structure of a dinuclear complex with bridging and non-bridging azido ligands, synthesized with picolinamide, a pyridine derivative with an amido group, suitable for the chelation of transition metals (Đaković *et al.*, 2008 ▸).
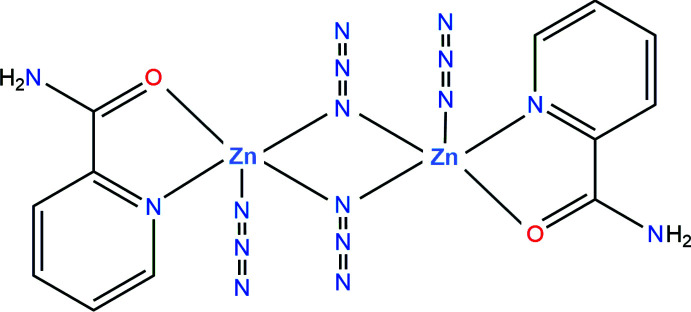


## Structural commentary   

**Figure 1:**
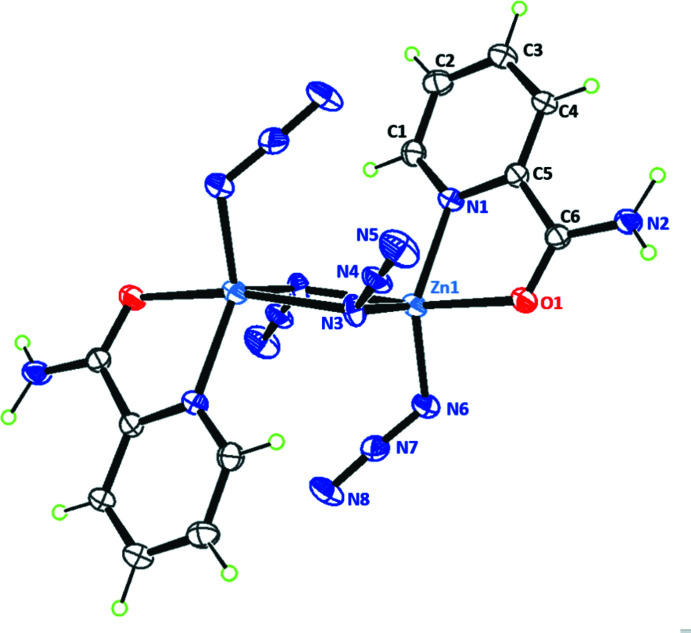
Mol­ecular structure of the title compound, showing 50% probability displacement ellipsoids for non-H atoms. Non-labelled atoms are generated by the symmetry operation 1 − *x*, 1 − *y*, 1 − *z*.

The dinuclear complex [Zn_2_(N_3_)_4_(pca)_2_], where pca is picolinamide (IUPAC name: pyridine-2-carboxamide), crystallizes in the triclinic space group *P* 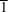, with the mol­ecule placed on the inversion centre (Fig. 1 ▸). The central [Zn_2_N_2_] core is thus planar by symmetry, with azido ligand N3/N4/N5 bridging the metal centres in the EO configuration. The double bridge is asymmetric, with Zn—N3 bond lengths of 2.057 (3) and 2.218 (3) Å (Table 1 ▸). These bond lengths are comparable to those observed in other Zn^2+^ complexes bearing Schiff bases (Ray *et al.*, 2012 ▸; Đaković *et al.*, 2015 ▸; Sheng *et al.*, 2014 ▸), and are in agreement with IR spectroscopy data (You *et al.*, 2009 ▸; Qian & You, 2011 ▸). The pca mol­ecule behaves as a κ^2^-*N*,*O*-chelating ligand, forming a common five-membered metallacycle. This mode of coordination is almost universally found in other complexes including pca as ligand: there are very few occurrences of κ^2^-*N*,*N*-pca ligands reported so far in the CSD. Finally, each Zn centre coordinates one terminal azido ion, N6/N7/N8, with the short distance Zn—N6 = 1.991 (4) Å. Both independent azido ligands are nearly linear, and the bridging azido has a bent coordination with the metal centre. In the dinuclear complex, the Zn⋯Zn separation is 3.2760 (11) Å.

**Figure 2:**
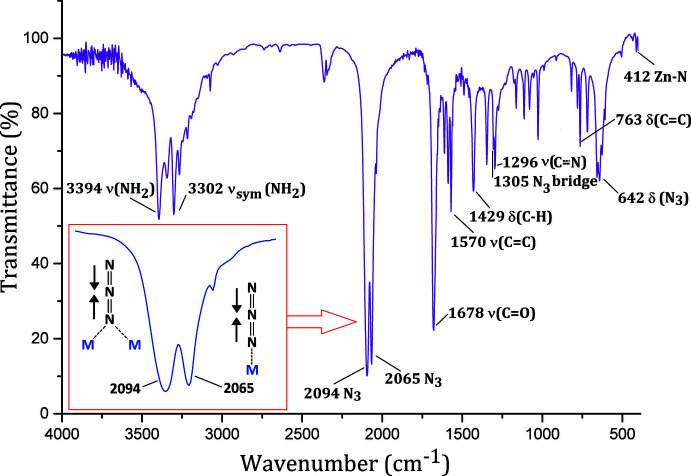
IR spectrum (KBr pellet) of the title complex, with assignment of the main bands. The inset is an expansion of the anti­symmetric stretching vibrations for azide groups.

The IR spectrum of the solid shows the stretching modes of coordinated pca ligands (Fig. 2 ▸). The band at 1678 cm ^1^ is assigned to the *ν*_C= O_ vibration, which is shifted towards lower energy because of the C=O bond lengthening upon coord­ination [C6=O1: 1.250 (4) Å]. In contrast, the N—H stretching band of the amide group is not displaced in comparison to the free ligand, indicating that the NH_2_ group does not coordinate to Zn^2+^ ions (Konidaris *et al.*, 2012 ▸). The medium intensity band at 1296 cm^−1^ can be attributed to the *ν*_C N_ vibration in the pyridyl ring. The most useful IR vibrations are those related to azido ligands, which are clearly split over two frequencies, at 2094 and 2065 cm^−1^ (Fig. 2 ▸, inset). Based on previous reports in the literature, the former can be assigned to bridging-EO azido ligands and the latter to terminal azido ligands (Đaković *et al.*, 2015 ▸; Forster & Horrocks, 1966 ▸). Similar intensities for these bands are in agreement with the X-ray structure. Finally, Zn—N vibrations give a low-intensity band at 412 cm^−1^ (Majumder *et al.*, 2006 ▸).

The resulting dinuclear complex has five-coordinate Zn^2+^ ions, for which the Addison geometric parameter is τ_5_ = 0.55, midway between an ideal square-pyramidal (τ_5_ = 0) and a trigonal–bipyramidal geometry (τ_5_ = 1; Addison *et al.*, 1984 ▸). The strain caused by the five-membered metallacycle formed by the pca ligand [bite angle: 77.87 (12)°], together with the geometric restraint imposed by the central [Zn_2_N_2_] ring [N3—Zn1—N3^i^ angle: 80.02 (12)°] account for the observed trigonal distortion. Such distortion has been observed in other similar dinuclear five-coordinate Zn^2+^ complexes bearing both terminal and bridging azido ligands: for nine complexes retrieved from the CSD, the Addison parameter ranges from τ_5_ = 0.40 (Sun & Wang, 2007 ▸) to τ_5_ = 0.93 (Wang *et al.*, 2004 ▸).

**Figure 3:**
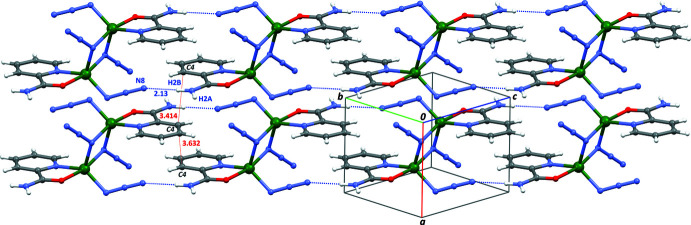
Part of the crystal structure of the title complex showing the arrangement of N—H⋯N hydrogen bonds. The proximity between π systems is reflected in the inter­molecular C4⋯C4 separations, as measured using *Mercury* (Macrae *et al.*, 2020 ▸; thin red lines): C4⋯C4^i^ = 3.632 Å, and C4^i^⋯C4^ii^ = 3.414 Å [symmetry codes: (i) 1 − *x*, 2 − *y*, −*z*; (ii) −1 + *x*, *y*, *z*]. The strongest inter­molecular hydrogen bond (Table 2 ▸, entry 2) is represented by blue dotted lines.

Non-covalent inter­molecular inter­actions are present in the crystal structure. Given that the NH_2_ groups in the pca ligands are not engaged in coordination, they form instead weak inter­molecular N—H⋯N hydrogen bonds with terminal N atoms of azide groups (Table 2 ▸). These bonds form a 2D framework parallel to plane (100) in the crystal. The mol­ecules are then arranged in such a way that pyridyl rings are stacked in the [100] direction, with an offset face-to-face arrangement characterized by centroid-to-centroid distances for pyridyl rings of 4.702 (3) and 5.141 (3) Å along a stack (Fig. 3 ▸).

## NMR measurements and chemical shift calculations   

Using DMSO-*d*_6_ solutions of the free ligand pca and the title complex, ^1^H and ^13^C-NMR spectra were recorded on a Bruker Avance III 500 MHz spectrometer. Computationally, the geometry for the complex was optimized with the BLYP functional (Becke, 1993 ▸) and the 6-31+G(2*d*,*p*) basis to correlate the experimental structural information, time-dependent DFT, and NMR chemical shift estimations. Bond lengths and angles are similar in the DFT-optimized structure and in the X-ray crystal structure, validating the correctness of the calculations (*GAUSSIAN*09; Frisch *et al.*, 2009 ▸). The shielding scales were converted to chemical shift scales by applying reference shielding of 32.0531 and 178.5112 ppm for ^1^H and ^13^C in TMS, respectively.

**Figure 4:**
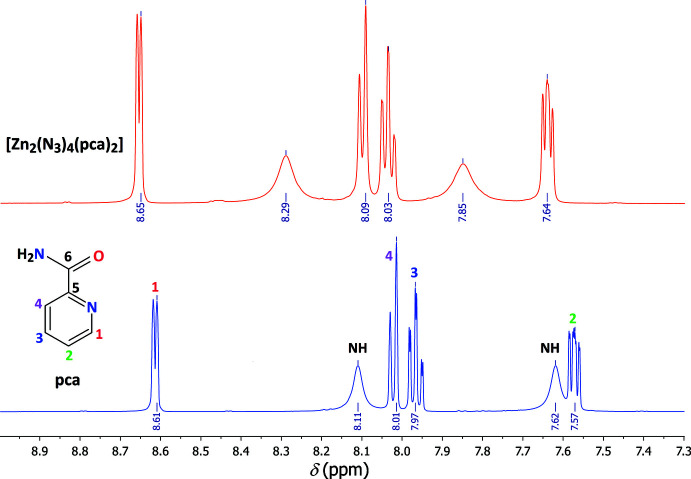
Experimental ^1^H-NMR spectra of pca (blue) and [Zn_2_(N_3_)_4_(pca)_2_] (red) in DMSO-*d*_6_. Chemical shifts and coupling constants are given in Table 3 ▸.

**Figure 5:**
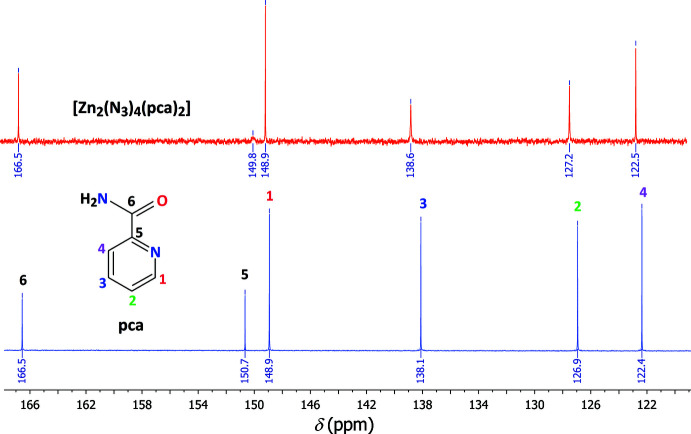
Experimental ^13^C-NMR spectra of pca (blue) and [Zn_2_(N_3_)_4_(pca)_2_] (red) in DMSO-*d*_6_. Chemical shifts are given in Table 3 ▸.

The ^1^H and ^13^C data of pca together with those of the complex are displayed in Table 3 ▸. Moreover, ^1^H and ^13^C chemical shifts were calculated, allowing the assignment of all signals in the experimental spectra (Figs. 4 ▸ and 5 ▸). The aromatic ^1^H spin systems are identified assuming doublet-like signals for H1 and H4, and triplet-like signals for H2 and H3. The presence of two NH broad signals with short relaxation times is due to the presence of the N and Zn atoms, which are more electronegative than H. The proton signals are slightly deshielded upon complexation, with the magnitude of deshielding decreasing while the distance from the metal centre increases. As seen in Fig. 4 ▸, the 3*d* ^10^ cation does not affect the position of the signals very much. The most affected signals are those corresponding to the amide NH groups, which are shifted by *ca* 0.2 ppm and broadened upon coordination. This behaviour is probably related to different hydrogen-bonding schemes involving the NH_2_ group: free pca is strongly stabilized in the solid state by 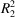
(8) ring motifs (Évora *et al.*, 2012 ▸), which are no longer present once the mol­ecule is coordinated to the metal centre. The small influence of the metal centres on NMR properties is confirmed by experimental ^13^C-NMR chemical shifts, which are almost identical for pca and the title complex (Fig. 5 ▸). However, a broadening is observed for the quaternary carbon atom C5, which is located in the close vicinity of the N and Zn sites, resulting in a very short relaxation time.

These data corroborate that proton chemical shifts for pca are only marginally affected by coordination to a diamagnetic metal centre as Zn^2+^. Very different spectra would be expected with paramagnetic centres, such as Mn^2+^, Co^2+^, or Cu^2+^. Most often, NMR spectra are difficult to inter­pret for these complexes, due to their broad and out of tune signals. However, our NMR data do not allow determination of whether the complex survives as a dimeric compound in solution, and whether the hydrogen bonding scheme observed in the crystal structure is retained in solution.

## Synthesis and crystallization   

An aqueous solution of pca (0.122 g, 1.0 mmol in 10 mL) was slowly poured onto an aqueous solution of Zn(SO_4_)·7H_2_O (0.287 g, 1.0 mmol in 10 mL) and an aqueous solution of NaN_3_ (0.130 g, 2.0 mmol in 5 mL). After one week at room temperature, colourless crystals formed in the mixture. Yield: 90%. Melting point: 471 K. The complex is soluble in water, DMSO, DMF and ethanol. IR data (cm^−1^, KBr pellet): 3394 (amide *ν*_N—H_), 3302 (amide *ν*_symN—H_), 2094, 2065 (ν_asymm_(N_3_)), 1678 (*ν*_C=O_), 1570 (*ν*_C C_), 1296 (*ν*_C N_). UV–Vis (λ_max_/nm, H_2_O, *ca* 10^−5^ *M*): 215 (π → π^*^), 264 (n → π^*^).

## Refinement   

Crystal data, data collection and structure refinement details are summarized in Table 4 ▸. All C-bound H atoms were placed in calculated positions and refined as riding on their carrier C atoms, while amide H atoms were found in a difference map and refined with free orientation. The geometry of the NH_2_ group was restrained with distance targets N—H = 0.87 (2) Å, and isotropic displacement parameters for these H atoms were calculated as *U*_iso_(H) = 1.2*U*_eq_(N2).

## Supplementary Material

Crystal structure: contains datablock(s) I, global. DOI: 10.1107/S2056989020016680/yz2003sup1.cif

Structure factors: contains datablock(s) I. DOI: 10.1107/S2056989020016680/yz2003Isup2.hkl

CCDC reference: 2052868

Additional supporting information:  crystallographic information; 3D view; checkCIF report

## Figures and Tables

**Table 1:** Selected geometric parameters (Å, °)

Zn1—N6	1.991 (4)	Zn1—O1	2.119 (3)
Zn1—N1	2.057 (3)	Zn1—N3^i^	2.218 (3)
Zn1—N3	2.057 (3)		
			
N6—Zn1—N1	133.42 (14)	N3—Zn1—O1	92.16 (12)
N6—Zn1—N3	112.66 (15)	N6—Zn1—N3^i^	94.82 (14)
N1—Zn1—N3	113.87 (13)	N1—Zn1—N3^i^	95.16 (13)
N6—Zn1—O1	98.33 (13)	N3—Zn1—N3^i^	80.02 (12)
N1—Zn1—O1	77.87 (12)	O1—Zn1—N3^i^	166.53 (12)

Symmetry code: (i) 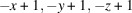.

**Table 2:** Hydrogen-bond geometry (Å, °)

*D*—H⋯*A*	*D*—H	H⋯*A*	*D*⋯*A*	*D*—H⋯*A*
N2—H2*A*⋯N5^ii^	0.85 (2)	2.41 (3)	3.184 (5)	151 (4)
N2—H2*B*⋯N8^iii^	0.88 (2)	2.13 (2)	2.994 (5)	166 (4)

Symmetry codes: (ii) 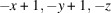; (iii) 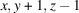.

**Table 3:** ^1^H-NMR (500 MHz) and ^13^C-NMR (125 MHz) chemical shifts, *δ* (ppm), and coupling constants *J*_H—H_ (Hz), for the ligand pca and the diamagnetic complex [Zn_2_(N_3_)_4_(pca)_2_], in DMSO-*d*_6_

	^1^H-NMR (experimental)	H⋯H coupling	^1^H-NMR (calculated)	^13^C-NMR (experimental)	^13^C-NMR (calculated)
Picolinamide	H4: 8.01	*d*, *J* = 7.8	H4: 8.18	C4: 122.36	C4: 127.02
	H3: 7.97	*td*, *J* = 7.6, 1.7	H3: 7.94	C3: 138.12	C3: 142.08
	H2: 7.57	*ddd*, *J* = 7.5, 4.8, 1.3	H2: 7.56	C2: 126.94	C2: 130.65
	H1: 8.61	*ddd*, *J* = 4.7	H1: 8.68	C1: 148.92	C1: 153.41
	NH_2_: 8.11, 7.62	*broad s*	NH_2_: 5.18, 7.66	C5: 150.66	C5: 155.63
				C6: 166.55	C6: 171.00
					
[Zn_2_(N_3_)_4_(pca)_2_]	H4: 8.09	*d*, *J* = 7.7	H4: 8.00	C4: 122.51	C4: 128.35
	H3: 8.03	*td*, *J* = 7.6	H3: 8.33	C3: 138.56	C3: 146.74
	H2: 7.64	*m*	H2: 7.92	C2: 127.24	C2: 134.07
	H1: 8.65	*d*, *J* = 4.6	H1: 9.08	C1: 148.93	C1: 154.42
	NH_2_: 8.29, 7.85	*broad s*	NH_2_: 6.84, 6.13	C5: 149.81	C5: 149.48
				C6: 166.53	C6: 170.84

**Table 4:** Experimental details

Crystal data	
Chemical formula	[Zn_2_(N_3_)_4_(C_6_H_6_N_2_O)_2_]
*M*_r_	543.12
Crystal system, space group	Triclinic, *P* 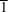
Temperature (K)	295
*a*, *b*, *c* (Å)	6.7689 (8), 8.3283 (10), 9.4835 (11)
α, β, γ (°)	69.942 (9), 75.447 (9), 75.901 (9)
*V* (Å^3^)	478.74 (10)
*Z*	1
Radiation type	Ag *K*α, λ = 0.56083 Å
μ (mm^−1^)	1.35
Crystal size (mm)	0.27 × 0.04 × 0.03

Data collection	
Diffractometer	Stoe Stadivari
Absorption correction	Multi-scan (*X-AREA*; Stoe & Cie, 2018 ▸)
*T*_min_, *T*_max_	0.426, 1.000
No. of measured, independent and observed [*I* > 2σ(*I*)] reflections	11210, 2088, 1344
*R*_int_	0.099
(sin θ/λ)_max_ (Å^−1^)	0.639

Refinement	
*R*[*F* ^2^ > 2σ(*F* ^2^)], *wR*(*F* ^2^), *S*	0.040, 0.078, 0.80
No. of reflections	2088
No. of parameters	151
No. of restraints	2
H-atom treatment	H atoms treated by a mixture of independent and constrained refinement
Δρ_max_, Δρ_min_ (e Å^−3^)	0.65, −0.52

Computer programs: *X-AREA* (Stoe & Cie, 2018 ▸), *SHELXT2018/2* (Sheldrick, 2015*a* ▸), *SHELXL2018/3* (Sheldrick, 2015*b* ▸), *ORTEP-3 for Windows* (Farrugia, 2012 ▸), *Mercury* (Macrae *et al.*, 2020 ▸) and *publCIF* (Westrip, 2010 ▸).

## supplementary crystallographic information

### Crystal data

**Table:**

[Zn_2_(N_3_)_4_(C_6_H_6_N_2_O)_2_]	*F*(000) = 272
*M**_r_* = 543.12	*D*_x_ = 1.884 Mg m^−^^3^
Triclinic, *P*1	Melting point: 471 K
*a* = 6.7689 (8) Å	Ag *K*α radiation, λ = 0.56083 Å
*b* = 8.3283 (10) Å	Cell parameters from 6594 reflections
*c* = 9.4835 (11) Å	θ = 2.5–22.2°
α = 69.942 (9)°	µ = 1.35 mm^−^^1^
β = 75.447 (9)°	*T* = 295 K
γ = 75.901 (9)°	Needle, colourless
*V* = 478.74 (10) Å^3^	0.27 × 0.04 × 0.03 mm
*Z* = 1	

### Data collection

**Table:**

Stoe Stadivari diffractometer	2088 independent reflections
Radiation source: Sealed X-ray tube, Axo Astix-f Microfocus source	1344 reflections with *I* > 2σ(*I*)
Graded multilayer mirror monochromator	*R*_int_ = 0.099
Detector resolution: 5.81 pixels mm^-1^	θ_max_ = 21.0°, θ_min_ = 2.5°
ω scans	*h* = −8→8
Absorption correction: multi-scan (X-AREA; Stoe & Cie, 2018)	*k* = −10→10
*T*_min_ = 0.426, *T*_max_ = 1.000	*l* = −11→12
11210 measured reflections	

### Refinement

**Table:**

Refinement on *F*^2^	Primary atom site location: dual
Least-squares matrix: full	Secondary atom site location: difference Fourier map
*R*[*F*^2^ > 2σ(*F*^2^)] = 0.040	Hydrogen site location: mixed
*wR*(*F*^2^) = 0.078	H atoms treated by a mixture of independent and constrained refinement
*S* = 0.80	*w* = 1/[σ^2^(*F*_o_^2^) + (0.0255*P*)^2^] where *P* = (*F*_o_^2^ + 2*F*_c_^2^)/3
2088 reflections	(Δ/σ)_max_ < 0.001
151 parameters	Δρ_max_ = 0.65 e Å^−^^3^
2 restraints	Δρ_min_ = −0.52 e Å^−^^3^
0 constraints	

### Fractional atomic coordinates and isotropic or equivalent isotropic displacement parameters (Å^2^)

**Table:**

	*x*	*y*	*z*	*U*_iso_*/*U*_eq_	
Zn1	0.71118 (8)	0.52257 (7)	0.37011 (6)	0.03385 (16)	
O1	0.7775 (4)	0.5513 (4)	0.1331 (3)	0.0381 (7)	
N1	0.7228 (5)	0.7831 (4)	0.2758 (4)	0.0297 (8)	
N2	0.8242 (6)	0.7454 (5)	−0.1008 (4)	0.0412 (9)	
H2A	0.859 (6)	0.663 (4)	−0.141 (5)	0.049*	
H2B	0.827 (7)	0.852 (3)	−0.164 (4)	0.049*	
N3	0.4244 (5)	0.4627 (5)	0.3951 (4)	0.0346 (8)	
N4	0.3234 (5)	0.5162 (4)	0.2967 (4)	0.0378 (9)	
N5	0.2268 (6)	0.5678 (5)	0.2013 (4)	0.0562 (12)	
N6	0.9229 (5)	0.3132 (5)	0.4351 (4)	0.0430 (9)	
N7	0.8669 (5)	0.1971 (5)	0.5432 (4)	0.0390 (9)	
N8	0.8183 (7)	0.0821 (5)	0.6474 (5)	0.0589 (12)	
C1	0.6936 (6)	0.8957 (6)	0.3551 (5)	0.0366 (10)	
H1	0.678297	0.853763	0.460923	0.044*	
C2	0.6855 (7)	1.0727 (6)	0.2841 (5)	0.0419 (11)	
H2	0.667083	1.147761	0.341574	0.050*	
C3	0.7048 (6)	1.1356 (6)	0.1285 (5)	0.0413 (11)	
H3	0.696926	1.254022	0.079321	0.050*	
C4	0.7364 (6)	1.0207 (5)	0.0448 (5)	0.0347 (10)	
H4	0.750748	1.060098	−0.061039	0.042*	
C5	0.7458 (6)	0.8456 (5)	0.1235 (4)	0.0290 (9)	
C6	0.7848 (6)	0.7035 (5)	0.0490 (5)	0.0303 (9)	

### Atomic displacement parameters (Å^2^)

**Table:**

	*U*^11^	*U*^22^	*U*^33^	*U*^12^	*U*^13^	*U*^23^
Zn1	0.0414 (3)	0.0265 (3)	0.0289 (3)	−0.0046 (2)	−0.0060 (2)	−0.0036 (2)
O1	0.0548 (19)	0.0256 (16)	0.0287 (16)	−0.0070 (14)	−0.0027 (13)	−0.0050 (13)
N1	0.0309 (18)	0.0261 (19)	0.0302 (19)	−0.0045 (15)	−0.0083 (14)	−0.0043 (15)
N2	0.066 (3)	0.028 (2)	0.027 (2)	−0.005 (2)	−0.0079 (18)	−0.0076 (17)
N3	0.044 (2)	0.043 (2)	0.0190 (18)	−0.0124 (18)	−0.0077 (16)	−0.0078 (16)
N4	0.049 (2)	0.024 (2)	0.038 (2)	−0.0150 (17)	−0.0050 (18)	−0.0025 (17)
N5	0.075 (3)	0.056 (3)	0.043 (2)	−0.015 (2)	−0.033 (2)	−0.003 (2)
N6	0.043 (2)	0.031 (2)	0.040 (2)	0.0021 (17)	−0.0037 (17)	−0.0019 (18)
N7	0.041 (2)	0.034 (2)	0.038 (2)	0.0014 (18)	−0.0099 (17)	−0.0098 (18)
N8	0.075 (3)	0.036 (2)	0.049 (3)	−0.012 (2)	−0.012 (2)	0.010 (2)
C1	0.046 (3)	0.036 (3)	0.030 (2)	−0.004 (2)	−0.0099 (19)	−0.012 (2)
C2	0.051 (3)	0.032 (3)	0.047 (3)	−0.009 (2)	−0.011 (2)	−0.015 (2)
C3	0.046 (3)	0.029 (2)	0.045 (3)	−0.009 (2)	−0.006 (2)	−0.007 (2)
C4	0.040 (2)	0.027 (2)	0.032 (2)	−0.0039 (19)	−0.0071 (19)	−0.0024 (19)
C5	0.027 (2)	0.028 (2)	0.030 (2)	−0.0026 (17)	−0.0039 (17)	−0.0070 (18)
C6	0.027 (2)	0.031 (2)	0.030 (2)	−0.0026 (18)	−0.0058 (17)	−0.0056 (19)

### Geometric parameters (Å, º)

**Table:**

Zn1—N6	1.991 (4)	N4—N5	1.151 (4)
Zn1—N1	2.057 (3)	N6—N7	1.197 (5)
Zn1—N3	2.057 (3)	N7—N8	1.159 (5)
Zn1—O1	2.119 (3)	C1—C2	1.389 (6)
Zn1—N3^i^	2.218 (3)	C1—H1	0.9300
O1—C6	1.250 (4)	C2—C3	1.370 (6)
N1—C5	1.339 (5)	C2—H2	0.9300
N1—C1	1.342 (5)	C3—C4	1.388 (6)
N2—C6	1.314 (5)	C3—H3	0.9300
N2—H2A	0.852 (19)	C4—C5	1.385 (5)
N2—H2B	0.884 (19)	C4—H4	0.9300
N3—N4	1.193 (4)	C5—C6	1.515 (5)
			
N6—Zn1—N1	133.42 (14)	N7—N6—Zn1	117.3 (3)
N6—Zn1—N3	112.66 (15)	N8—N7—N6	178.1 (5)
N1—Zn1—N3	113.87 (13)	N1—C1—C2	122.1 (4)
N6—Zn1—O1	98.33 (13)	N1—C1—H1	119.0
N1—Zn1—O1	77.87 (12)	C2—C1—H1	119.0
N3—Zn1—O1	92.16 (12)	C3—C2—C1	119.2 (4)
N6—Zn1—N3^i^	94.82 (14)	C3—C2—H2	120.4
N1—Zn1—N3^i^	95.16 (13)	C1—C2—H2	120.4
N3—Zn1—N3^i^	80.02 (12)	C2—C3—C4	119.3 (4)
O1—Zn1—N3^i^	166.53 (12)	C2—C3—H3	120.3
C6—O1—Zn1	114.5 (3)	C4—C3—H3	120.3
C5—N1—C1	118.1 (4)	C5—C4—C3	118.1 (4)
C5—N1—Zn1	116.5 (3)	C5—C4—H4	121.0
C1—N1—Zn1	125.2 (3)	C3—C4—H4	121.0
C6—N2—H2A	117 (3)	N1—C5—C4	123.1 (4)
C6—N2—H2B	125 (3)	N1—C5—C6	112.3 (3)
H2A—N2—H2B	117 (4)	C4—C5—C6	124.6 (4)
N4—N3—Zn1	123.8 (3)	O1—C6—N2	122.8 (4)
N4—N3—Zn1^i^	121.5 (3)	O1—C6—C5	118.5 (4)
Zn1—N3—Zn1^i^	99.98 (12)	N2—C6—C5	118.7 (4)
N5—N4—N3	179.7 (5)		
			
C5—N1—C1—C2	0.4 (6)	C3—C4—C5—N1	1.1 (6)
Zn1—N1—C1—C2	−174.2 (3)	C3—C4—C5—C6	−178.3 (4)
N1—C1—C2—C3	1.0 (6)	Zn1—O1—C6—N2	179.9 (3)
C1—C2—C3—C4	−1.3 (6)	Zn1—O1—C6—C5	−0.3 (4)
C2—C3—C4—C5	0.4 (6)	N1—C5—C6—O1	4.7 (5)
C1—N1—C5—C4	−1.4 (6)	C4—C5—C6—O1	−175.8 (4)
Zn1—N1—C5—C4	173.6 (3)	N1—C5—C6—N2	−175.5 (4)
C1—N1—C5—C6	178.0 (3)	C4—C5—C6—N2	4.0 (6)
Zn1—N1—C5—C6	−6.9 (4)		

Symmetry code: (i) −*x*+1, −*y*+1, −*z*+1.

### Hydrogen-bond geometry (Å, º)

**Table:**

*D*—H···*A*	*D*—H	H···*A*	*D*···*A*	*D*—H···*A*
N2—H2*A*···N5^ii^	0.85 (2)	2.41 (3)	3.184 (5)	151 (4)
N2—H2*B*···N8^iii^	0.88 (2)	2.13 (2)	2.994 (5)	166 (4)
C1—H1···N3^i^	0.93	2.69	3.243 (5)	119
C4—H4···N8^iii^	0.93	2.64	3.541 (6)	165

Symmetry codes: (i) −*x*+1, −*y*+1, −*z*+1; (ii) −*x*+1, −*y*+1, −*z*; (iii) *x*, *y*+1, *z*−1.
